# Role of Specialty Drugs in Rising Drug Prices for Medicare Part D

**DOI:** 10.1001/jamahealthforum.2024.1188

**Published:** 2024-05-24

**Authors:** Tamara B. Hayford

**Affiliations:** 1 Congressional Budget Office, Washington, DC

## Abstract

**Importance:**

Prices for brand-name drugs affect both federal spending and out-of-pocket liability for Medicare Part D enrollees.

**Objective:**

To examine how prices for brand-name drugs, net of rebates and discounts, have changed from 2010 to 2019 and to examine the role of specialty drugs in those changes.

**Design, Setting, and Participants:**

This study involved a descriptive analysis of prescription drug spending and prices between 2010 and 2019. The universe of prescription drug event data from those years were combined with confidential data from the Centers for Medicare & Medicaid Services on rebates and discounts that manufacturers and pharmacies pay to Medicare Part D plans to calculate rebate percentages, net spending, and net prices at the drug level. Specialty drugs were identified using information from IQVIA, allowing for a stratified analysis by specialty status. Data were analyzed from March 2019 to March 2024.

**Main Outcomes and Measures:**

Average prices (net of rebates and discounts in 2019 US dollars) and average annual price growth for brand-name prescription drugs, overall and separately for specialty and nonspecialty drugs.

**Results:**

Average net prices for brand-name drugs doubled from 2010 to 2019 (from $167 to $370). Growth in specialty drug prices was an underlying factor in those increases: average annual price growth was 13.2% for specialty drugs compared with 2.6% for nonspecialty drugs. Price growth for specialty drugs over the decade was smaller than what the Congressional Budget Office reported for the 2010 to 2015 period (increase of 22.3% per year vs 4.5% per year for nonspecialty drug prices), suggesting that price growth slowed after 2015. Drugs that treat hepatitis C contributed to that difference because prices for those drugs were initially high and then subsequently fell. Absent those drugs, price growth for specialty drugs averaged 18.1% in the first half of the decade and 6.9% in the second half.

**Conclusions and Relevance:**

Results of this study show that prices for specialty drugs have continued to increase over time in the Medicare Part D program, which contributes to high out-of-pocket liability for users of those drugs in addition to US federal budgetary expenditures.

## Key Points

**Question:**

How have prices for specialty drugs affected price growth in the Medicare Part D program?

**Findings:**

In this study of prescription drug spending and prices between 2010 and 2019, average prices grew considerably faster for specialty brand-name drugs than for nonspecialty drugs in the Medicare Part D program. Price growth for specialty drugs slowed in the second half of the period, and price changes for the class of drugs that treat hepatitis C may have been a factor, as they had high prices initially followed by subsequent price reductions.

**Meaning:**

Part D enrollees who use specialty drugs experienced increasing prices, and those without cost-sharing assistance experienced higher out-of-pocket liability.

## Teaser

This study examines how prices for brand-name drugs, net of rebates and discounts, have changed from 2010 to 2019 as well as the role of specialty drugs in those changes.

## Introduction

Prices for prescription drugs receive substantial attention from policymakers, as evidenced by the 2022 reconciliation act and other legislative efforts to reduce drug prices.^1^ The average price of a brand-name prescription in Medicare Part D, net of rebates and discounts, more than doubled from $167 in 2010 to $370 in 2019 (in 2019 US dollars)—for an annual average increase of nearly 10% per year. The rising prominence of specialty drugs—which treat complex conditions, require special handling, and have high prices—was a driving factor in that increase. In 2010, brand-name specialty drugs accounted for 17% of net spending on brand-name drugs in Medicare Part D. By 2019, that percentage was 54% (Table 1).

**Table 1.** Change in the Average Net Price, Share of Spending, and Rebate Percentage of a Brand-Name Prescription for Specialty and Nonspecialty Drugs in Medicare Part D, 2010-2019^a^

Measurement	2010	2019	Average annual change, %
**Average net price per prescription, 2019 US $**			
All brand-name drugs	167	370	9.2
Brand-name specialty drugs			
All drugs	1400	4260	13.2
Older drugs^b^	1400	3410	10.4
New drugs^c^	NA	5450	NA
Brand-name nonspecialty drugs			
All drugs	142	179	2.6
Older drugs^b^	142	164	1.7
New drugs^c^	NA	215	NA
**Share of brand-name drug spending, %**			
All brand-name drugs	100	100	NA
Brand-name specialty drugs			
All drugs	16.8	54.0	13.8
Older drugs^b^	16.8	25.3	4.6
New drugs^c^	NA	28.7	NA
Brand-name nonspecialty drugs			
All drugs	83.2	46.0	−6.4
Older drugs^b^	83.2	30.5	−10.6
New drugs^c^	NA	15.5	NA
**Rebate, %** ^d^			
Brand-name specialty drugs, mean (IQR)^e^	2.6 (0-0.2)	14.2 (4.9-10.4)	20.8
Brand-name nonspecialty drugs, mean (IQR)^e^	17.4 (0-0.7)	54.4 (4.9-20.4)	13.5

Abbreviation: NA, not applicable.

^a^ Spending amounts are adjusted for inflation using the Personal Consumption Expenditures Price Index as reported by the Bureau of Economic Analysis.

^b^ Older drugs are those on the market by 2010.

^c^ Newer drugs are new molecules approved by the US Food and Drug Administration from 2011 to 2019.

^d^ The rebate percentage is the sum of negotiated rebates between plans and manufacturers, negotiated discounts between plans and pharmacies, and the statutory discount that manufacturers pay plans for spending on their drugs in the coverage gap divided by gross payments to pharmacies.

^e^ IQR of rebate percentages is computed from percentiles of drug-level rebate percentages.

## Methods

This study relied on 2 datasets: (1) the prescription drug event files from Medicare Part D and (2) a confidential dataset on the rebates and discounts that Part D plans receive from manufacturers and pharmacies obtained from the Centers for Medicare & Medicaid Services. CBO has statutory authority to receive these confidential rebate and discount data from the Centers for Medicare & Medicaid Services.^2^ I combined those datasets at the national drug code level and calculated total spending over the 2010 to 2019 period for each drug.^3^ To abstract from any trends in filling longer prescriptions over the period, I also calculated the number of standardized 30-day prescriptions for each drug over that period.

Net spending and prices represent gross payments to pharmacies, net of rebates and discounts, which comprise negotiated rebates between plans and manufacturers, negotiated discounts between plans and pharmacies, and the statutory discount that manufacturers pay plans for spending on their drugs in the coverage gap. The coverage gap discount equaled 50% from 2011 to 2018 and increased to 70% in 2019.^4^ All dollar amounts were adjusted for inflation using the Personal Consumption Expenditures Price Index as reported by the Bureau of Economic Analysis.^5^

This analysis was stratified by specialty status. I identified specialty drugs using a list of specialty drugs on the market in 2015 that was purchased from IQVIA and updated it by applying IQVIA’s definition of specialty drugs to all new molecules and new combinations of active ingredients that entered the market between 2016 and 2019. Specialty drugs treat chronic, complex, or rare conditions and have at least 4 of 7 other key characteristics.^6^ See the eMethods in Supplement 1 for more detail on the data construction.

This analysis extends a prior analysis to examine the role of specialty drugs in driving growth in spending and average prices for brand-name drugs from 2010 to 2019.^7^ Higher prices for new drugs and price growth for older drugs are 2 components of overall price growth. New drugs often launch at higher prices, and prices for older drugs often increase over time. New drugs are defined as new molecular entities that entered the market after 2010. I used the novel drug approval lists for years 2011 to 2019,^8^ published by the US Food and Drug Administration, to differentiate between new drugs and new combinations of existing drugs, which were not classified as new drugs in this analysis.

This analysis was not submitted for institutional review board approval because it used deidentified data and did not constitute human participant research. It follows the Strengthening the Reporting of Observational Studies in Epidemiology (STROBE) reporting guidelines for cross-sectional studies.

## Results

Net spending on specialty brand-name drugs was $46.8 billion in 2019—5 times greater than the $9.4 billion spent in 2010. One-third of that increase occurred after 2015 when net spending on specialty brand-name drugs was $33.6 billion in 2019 US dollars.^9^ Meanwhile, specialty drugs represented only 0.5% of prescriptions over that period.

The underlying price growth for specialty drugs contributed to prices for brand-name drugs growing by an average of 9.2% per year from 2010 to 2019. The average net price of a specialty brand-name drug increased by 13.2% per year over that period, compared with 2.6% per year for nonspecialty drugs (Table 1). The majority of overall price growth can be attributed to price growth for drugs on the market in 2010: that growth represented 70.3% of price growth for specialty brand-name drugs and 59.5% for nonspecialty drugs. The remaining price growth is attributable to drugs that became available after 2010.

Growth in the rebates and discounts that Part D plans receive from manufacturers and pharmacies influence growth in net prices, allowing them to grow more slowly than wholesale prices.^10^ The rebate percentage grew considerably for both types of drugs, reaching 14.2% of gross spending for specialty drugs and 54.4% for nonspecialty drugs. Those averages mask considerable variation. The rebate distribution is skewed, with plans receiving large rebates for a relatively small fraction of drugs. For both specialty and nonspecialty drugs, the overall fraction of gross spending attributable to rebates and discounts is larger than the 75th percentile of the rebate distribution among all drugs.

The Congressional Budget Office reported faster average annual price growth for the 2010 to 2015 period: 22.3% for specialty drugs and 4.5% for nonspecialty drugs. For specialty drugs, only 55.3% of price growth was attributable to price growth for drugs that were on the market in 2010; 85.7% of price growth for nonspecialty drugs was attributable to older drugs.^7^ This suggests that drugs available after 2010 played a larger role for specialty drugs in the first half of the decade, while new drugs played a smaller role for nonspecialty drugs.

While price growth for specialty drugs appears to be slowing considerably, that is influenced by the trajectory of prices for hepatitis C antiviral drugs. Those drugs successfully treat a large percentage of patients, and their prices were initially very high but rapidly fell because competing products entered the market very quickly.^11^ When hepatitis C antiviral drugs are excluded, the slowdown for specialty drugs is less pronounced. Price growth averaged 18.1% per year in the first half of the decade, compared with 6.9% in the second half (Table 2); including those drugs, average price growth was 22.3% and 2.7%, respectively. Therefore, the marked slowdown may not continue. Future price growth will reflect new drugs and new competitors entering the market.

**Table 2.** Average Annual Net Price Growth for Specialty Brand-Name Drugs With and Without Drugs That Treat Hepatitis C, 2010-2019

Analysis	%		
	2010-2019	2010-2015	2015-2019
Original	13.2	22.3	2.7
Excluding drugs that treat hepatitis C	13.0	18.1	6.9

Rising prices for specialty drugs can affect spending for their users. Annual net spending on specialty brand-name drugs per person who used such drugs rose from $13 010 in 2010 to $35 550 in 2019 (Figure). That increase was larger among people without cost-sharing assistance through either the low-income subsidy program or an employer retiree plan: from $10 090 to $40 780. Average annual out-of-pocket costs rose from $2010 to $3340 for those enrollees.

**Figure.**
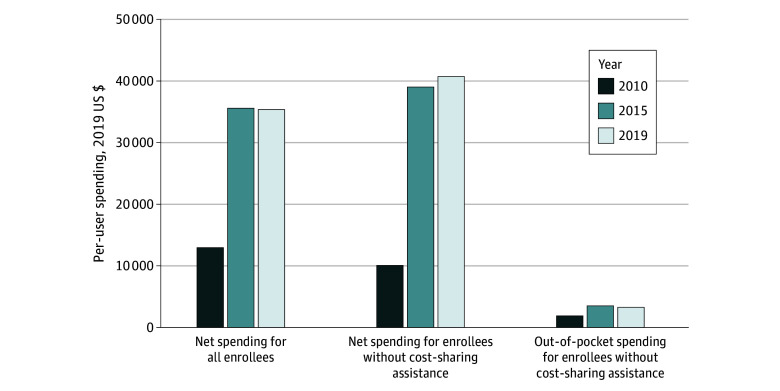
Net and Out-of-Pocket Spending on Brand-Name Specialty Drugs Among Medicare Part D Enrollees Who Used Such Drugs in 2010, 2015, and 2019 Spending amounts are adjusted for inflation using the Personal Consumption Expenditures Price Index as reported by the Bureau of Economic Analysis. Cost-sharing assistance is defined as cost-sharing subsidies provided through the low-income subsidy program or enrollment in an employer retiree plan.

## Discussion

Prices for brand-name specialty drugs in Medicare Part D tripled from $1400 in 2010 to $4260 in 2019, contributing to spending growth for specialty drugs. Growth in prices and spending slowed somewhat in the second half of the decade. These findings differ somewhat from other findings that increases in use are driving higher spending.^12^ Those differences may stem from analyses of different measures of use (percentage of prescriptions vs percentage of enrollees) or from focusing on a different subset of drugs (specialty drugs vs ultra-expensive drugs). However, the findings that growth slowed in the second half of the decade are largely consistent with other findings that nationwide net prices for drugs on the market by 2007 held steady after 2015. Slower price growth in the study by Hernandez et al^10^ is likely associated with the restriction to drugs on the market in 2007 and the inclusion of Medicaid’s statutory discounts.

It is perhaps surprising that per-enrollee spending in Part D over that period was relatively stable, by contrast.^13^ One key factor in that stability was a substantial increase in the use of generic drugs, which increased from 72% of dispensed prescriptions in 2009 to 90% by 2018.

### Limitations

The study has 2 key limitations. First, the analysis assessed only the prices for brand-name prescription drugs and did not consider the benefits of the drugs or compare the benefits with the prices. Second, it is a retrospective analysis that may not be predictive of future price growth. In particular, new policies from the 2022 reconciliation act may play a large role in determining the evolution of prices for brand-name drugs going forward. That will have implications for the financial burden of the Part D program on the federal budget in addition to the financial cost of those drugs for the people who use them.

## Conclusions

Results of this study show that prices for specialty drugs have continued to increase over time, which contributes to high out-of-pocket liability for users of those drugs in addition to federal budgetary expenditures.

## Notes

Supplement 1. eMethods

Supplement 2. Data Sharing Statement
