# Supplementary material for: Role of Specialty Drugs in Rising Drug Prices for Medicare Part D
Source: JAMA Health Forum. 2024 May 24;5(5):e241188. doi: 10.1001/jamahealthforum.2024.1188 (PMC11127124; doi:10.1001/jamahealthforum.2024.1188)
Supplement: Supplement 1. — eMethods [file jamahealthforum-e241188-s001.pdf]

Supplemental Online Content

Hayford TB. Role of specialty drugs in rising drug prices for Medicare Part D. *JAMA Health Forum*. 2024;5(5):e241188. doi:10.1001/jamahealthforum.2024.1188

eMethods

This supplemental material has been provided by the authors to give readers additional information about their work.

© 2024 Hayford T. *JAMA Health Forum.*

eMethods

This analysis is primarily based on two data sets: the prescription drug event (PDE) files for the Medicare Part D program and confidential data on the rebates and discounts that Part D plans receive from drug manufacturers and pharmacies. The PDE data contain a listing of all prescriptions that Part D enrollees purchased from pharmacies using their Part D plan. The data report a variety of information about the amounts paid by different entities (such as the beneficiary, plan, and cost-sharing subsidy) and other details about the prescription. For the purposes of this analysis, the key data fields are the total amount paid to the pharmacy, the number of days supplied in the prescription, and the drug's national drug code (NDC). NDC codes, which the Food and Drug Administration assigns, can be used to identify the manufacturer, chemical entity dosage form, route of administration, strength, and package size for a prescription drug. The data on rebates and discounts report—for each NDC code—the total rebate amount that drug manufacturers pay to Part D plans and the total amount of discounts that pharmacies pay to plans. The Centers for Medicare and Medicaid Services provided both data sets for calendar years 2010 to 2019.

Observations in the two data sets are summed at the NDC-year level, and then the two data sets are merged at the NDC level. Before that summation, the number of standardized 30-day prescriptions are calculated for each prescription in the PDE data to facilitate calculating standardized prices that are not affected by changes in prescribing patterns between 30-day and 90-day prescriptions. Total net spending for each NDC is calculated as the total amount paid to the pharmacy minus the sum of rebates and discounts paid to plans. Average net prices are calculated as total net spending divided by the number of standardized 30-day prescriptions.

The analysis is limited to brand-name drugs and stratified by specialty status. Specialty drugs were identified using a definition developed by IQVIA. According to that definition, specialty drugs are those that treat chronic, complex or rare conditions and have at least four of seven other key characteristics. The seven characteristics are: costs at least $6,000 per year; be initiated or maintained by a specialist; be administered by a health care professional; require special handling in the supply chain; be associated with a patient payment assistance program; be distributed through nontraditional channels (such as a specialty pharmacy); or require monitoring or counseling, either because of significant side effects or because of the type of disease being treated.

A list of specialty drugs on the market in 2015 was purchased from IQVIA. The list was updated for new drugs that entered the market from 2016 to 2019 using IQVIA's definition. Medicare Part D spending data were used to identify whether each new drug met the cost criterion, and internet research was used to identify whether the seven characteristics (other than costs) applied to each new drug. Specialty drugs are those for which at least four of these seven criterion were satisfied. Most cases were not borderline decisions; in other words, the number of criteria met was less than four or greater than four. Out of 182 drugs newly marketed between 2016 and 2019, 72 met three or fewer criteria, and 91 met five or more criteria; only 19 drugs met four criteria of the seven. Additional verification, such as checking whether drugs that treat the same condition were on the IQVIA list of specialty drugs in 2015 and additional internet research, was done for drugs that met three to five criteria to ensure accurate categorization. Details for how each criterion was considered follows:

1. The drug costs more than $6,000 per year: We used the Medicare Part D spending dashboard (https://www.cms.gov/Research-Statistics-Data-and-Systems/Statistics-Trends-and-Reports/Information-on-Prescription-Drugs/MedicarePartD) to assess whether average spending per beneficiary exceeded $6,000.
2. They are initiated (that is, prescribed) or maintained (that is, monitored) by a specialist: We used the Mayo Clinic website and NIH Rare Disease Database to determine whether the disease treated qualifies as a rare disease or is a type of cancer, whether patients are recommended to visit a specialist after receiving a diagnosis or to receive a diagnosis, or whether the disease is a progressive or severe disease for which other drug treatments may have failed or may not be suitable for progressive stages of the disease.
3. They are administered by a health care professional: We used the discussion of "Proper Use" on the Mayo Clinic website to determine whether patients could be trained to take the drug at home; drugs for which the only option is for a health care provider to administer the drug meet this criteria.

© 2024 Hayford T. *JAMA Health Forum.*

4. They require special handing in the supply chain: A drug meets this criterion if it must be shipped frozen or refrigerated according to the FDA Labels website, if it is a chemotherapy medication according to the Mayo Clinic website, or if it is biohazardous according to the National Institute for Occupational Safety and Health's NIOSH List of Antineoplastic and Other Hazardous Drugs in Health Care Settings, 2020.
5. They are associated with a patient payment assistance program: A drug was counted as having a patient assistance program if it was listed as having a program that allowed the drug to be purchased at little or no cost (that is, not a cost-sharing assistance program) in at least two of the following three databases:
    a. NeedyMeds: https://www.needymeds.org
    b. Medicare: https://www.medicare.gov/plan-compare/#/pharmaceutical-assistance-
    c. GoodRx: https://www.goodrx.com
6. They are distributed through nontraditional channels (such as a specialty pharmacy): A drug was counted as meeting this criterion if it was considered a specialty drug by at least two out of the following large pharmacy or insurance companies: CVS/Caremark, Cigna/Express Scripts, and OptumRx. If a drug is listed as being distributed through a specialty pharmacy in those three sites, but it is listed on the manufacturer website as only being available through specialty pharmacies, it is also counted as meeting this criterion.
7. They require monitoring or counseling either because of significant side effects or because of the type of disease being treated: A drug was counted as meeting this criterion if it was specified as needing additional testing during treatment on either the FDA Labels website or the Mayo Clinic website. Additional testing must be required for all patients taking the drug (not just certain subsets) in order for this criterion to be met.

© 2024 Hayford T. *JAMA Health Forum.*
