# Supplementary material for: Role of Specialty Drugs in Rising Drug Prices for Medicare Part D
Source: JAMA Health Forum. 2024 May 24;5(5):e241188. doi: 10.1001/jamahealthforum.2024.1188 (PMC11127124; doi:10.1001/jamahealthforum.2024.1188)
Supplement: Supplement 2. — Data Sharing Statement [file jamahealthforum-e241188-s002.pdf]

Data Sharing Statement

Hayford. Role of Specialty Drugs in Rising Drug Prices for Medicare Part D. *JAMA Health Forum*. Published May 24, 2024. doi:10.1001/jamahealthforum.2024.1188

Data
Data available: No

Additional Information
Explanation for why data not available: Components of the data are confidential and proprietary information.
